# Profiling Inflammatory Biomarkers following Curcumin Supplementation: An Umbrella Meta-Analysis of Randomized Clinical Trials

**DOI:** 10.1155/2023/4875636

**Published:** 2023-01-16

**Authors:** Navid Naghsh, Vali Musazadeh, Omid Nikpayam, Zeynab Kavyani, Amir Hossein Moridpour, Fatemeh Golandam, Amir Hossein Faghfouri, Alireza Ostadrahimi

**Affiliations:** ^1^Department of Pharmacy, Shahid Sadoughi University of Medical Sciences, Yazd, Iran; ^2^Student Research Committee, Faculty of Nutrition and Food Science, Tabriz University of Medical Sciences, Tabriz, Iran; ^3^Department of Nutritional Sciences School of Health, Golestan University of Medical Sciences, Gorgan, Iran; ^4^Department of Pharmacy, Mashhad University of Medical Science, Mashhad, Iran; ^5^Nutrition Research Center, Tabriz University of Medical Sciences, Tabriz, Iran; ^6^Maternal and Childhood Obesity Research Center, Urmia University of Medical Sciences, Urmia, Iran; ^7^Department of Clinical Nutrition Faculty of Nutrition, Tabriz University of Medical Sciences, Tabriz, Iran

## Abstract

**Objective:**

Several meta-analyses have shown that curcumin can reduce inflammatory biomarkers, but the findings are inconsistent. The objective of the present umbrella meta-analysis was to provide a more accurate estimate of the overall effects of curcumin on inflammatory biomarkers.

**Methods:**

The following international databases were systematically searched until March 20, 2022: PubMed, Scopus, Embase, Web of Science, and Google Scholar. A random-effects model was applied to evaluate the effects of curcumin on inflammatory biomarkers. Meta-analysis studies investigating the effects of curcumin supplementation on inflammatory biomarkers with corresponding effect sizes (ES) and confidence intervals (CI) were included in the umbrella meta-analysis. GRADE (Grading of Recommendations Assessment, Development, and Evaluation) was used to evaluate the certainty of evidence.

**Results:**

A meta-analyses of ten studies with 5,870 participants indicated a significant decrease in C-reactive protein (CRP) (ES = −0.74; 95% CI: −1.11, −0.37, *p* < 0.001; I^2^ = 62.1%, *p*=0.015), interleukin 6 (IL-6) (ES = −1.07; 95% CI: −1.71, −0.44, *p* < 0.001; I^2^ = 75.6%, *p* < 0.001), and tumour necrosis factor *α* (TNF-*α*) levels (ES: −1.92, 95% CI: −2.64, −1.19, *p* < 0.0; I^2^ = 18.1%, *p*=0.296) following curcumin supplementation. Greater effects on CRP and TNF-*α* were evident in trials with a mean age >45 years and a sample size >300 participants.

**Conclusion:**

The umbrella of meta-analysis suggests curcumin as a promising agent in reducing inflammation as an adjunctive therapeutic approach in diseases whose pathogenesis is related to a higher level of inflammatory biomarkers.

## 1. Introduction

Inflammation is a physiological response of the immune system induced by various harmful factors such as pathogens, injury, or damaged cells to restore body function [1, 2]. Chronic inflammation is typically characterized by a high number and excessive activation of innate immune cells in tissues and increased release of inflammatory mediators and chemokines at local and systemic levels. In the context of inflammation, interleukin 6 (IL-6) and tumor necrosis factor (TNF-*α*) are two cytokines that are released in significant amounts. They have been proven to be powerful inducers of C-reactive protein (CRP), which is a major acute-phase reactant produced mostly in the liver and strongly associated with metabolic diseases. It has been widely recognized that autoimmune diseases, atherosclerosis, asthma, type 2 diabetes (T2D), and other conditions can be caused by chronic inflammation [3–5].

A wide range of pharmacotherapies can improve inflammatory biomarkers, but they are also known to cause complications and side effects. Hence, nutraceutical therapies such as dietary supplements can be considered adjunctive or alternative treatments that can be utilized as anti-inflammatory agents [6–8]. Curcumin is remarkable bioactive polyphenol extracted from the rhizome of turmeric (Curcuma longa) [9, 10]. Curcumin has a wide range of medicinal effects such as hepatoprotective, antimicrobial, anti-inflammatory, antioxidant, and antitumor activities [11, 12].

The anti-inflammatory feature of curcumin is mediated by several pathways. Intestinal alkaline phosphatase, an endogenous antioxidant and anti-inflammatory enzyme, is upregulated by curcumin. Moreover, curcumin modulates inflammatory markers through nuclear factor-erythroid factor 2-related factor 2 (NRF2*∗*)-Keap1 regulatory pathway. The anti-inflammatory and antioxidant effects of Nrf2 in response to stress conditions have been investigated in several studies [13, 14]. Given these effects, curcumin can have regulatory effects on the level of inflammatory biomarkers such as CRP, IL-6, and TNF-*α* [14–17].

Despite promising results, some studies have found that curcumin supplementation has no considerable efficacy on inflammatory markers [18–20]. Taking into account of inconsistency, the current umbrella of meta-analysis was designed to reevaluate the anti-inflammatory effects of curcumin.

## 2. Methods

According to the guiding principle of the Preferred Reporting Items for Systematic Reviews and Meta-analysis (PRISMA), this study was carried out and reported [21]. The protocol of this study has been registered in the International Prospective Register of Systematic Reviews (PROSPERO) under number CRD42022323546.

### 2.1. Search Strategy and Study Selection

Relevant studies published on international scientific databases including PubMed, Scopus, Embase, Web of Science, and Google Scholar were searched until March 20, 2022. The search strategy was developed using the following MeSH terms and keywords: (“curcumin” OR “curcuminoid” OR “turmeric”) AND (“C-Reactive Protein” OR “crp” OR “hs-crp” OR “high sensitivity C-reactive protein” OR “Tumor Necrosis Factor-alpha” OR “tumor necrosis factor-*α*” OR “tnf-alpha” OR “tnf-*α*” OR “Interleukin-6” OR “IL-6” OR “inflammation”) AND (“systematic review” OR “meta-analysis”) The search strategy is shown in Suppl.  Table 1. To increase the sensitivity of the search strategy, the wild-card “*∗*” term was used. Also, the articles were limited to English.

### 2.2. Inclusion and Exclusion Criteria

Meta-analysis studies investigating the effects of curcumin supplementation on inflammatory biomarkers including CRP, IL-6, and TNF-*α* with corresponding effect sizes (ES) and confidence intervals (CI) were included in the umbrella meta-analysis. In addition, the following studies were excluded: in vitro, in vivo, and ex vivo studies; case reports; observational studies; quasi-experimental studies; controlled clinical trials; and studies with the lowest quality score.

### 2.3. Methodological Quality Assessment

A Measurement Tool to Assess Systematic Reviews (AMSTAR) 2 questionnaire was utilized by two independent researchers (VM and ZK) to assess the methodological quality of included meta-analyses of randomized controlled trials (RCTs) [22]. Disagreements were resolved by consensus with a third researcher (AO). The instrument (AMSTAR 2) retains 10 of the original domains and has 16 items in total. The questionnaire consists of 16 questions asking the reviewers to answer “yes” or “partial yes” or “no” or “no meta-analysis”. The AMSTAR 2 checklist was categorized into “critically low quality,” “low quality,” “moderate quality,” and “high quality.”

#### 2.3.1. Grading of the Evidence

We evaluated the overall strength and quality of evidence using the GRADE tool based on the Cochrane Handbook of systematic reviews of interventions. This tool consists of five factors: bias risk, consistency of results, directness, precision, and publication bias. GRADE results were categorized as “high,” “medium,” “low,” and “very low.” When one of the above factors is not met, the quality of a level decreases [23].

### 2.4. Study Selection and Data Extraction

Two independent reviewers (VM and AHM) screened the articles based on the eligibility criteria. In the first step, the title and abstract of the articles were reviewed. Then, the full-text of relevant articles was assessed to ascertain study suitability for inclusion in the umbrella meta-analysis. Any disagreements were resolved through the consensus with the senior author (AO).

The first authors' name, publication year, sample size, study location, dose and duration range of supplementation, ESs, and CIs for CRP, IL-6, and TNF-*α* were extracted from the selected meta-analyses.

### 2.5. Data Synthesis and Statistical Analysis

To estimate the pooled effect size, ESs and the corresponding CIs were utilized. I^2^ statistic and Cochrane's *Q*-test were applied to detect heterogeneity, and I^2^ value > 50% or *p* < 0.1 for *Q*-test was considered as meaningful heterogeneity among studies. The restricted maximum likelihood method was carried out to conduct statistical analysis based on random-effects model. Based on predetermined variables consisting of duration of intervention, mean age of participants, dose of curcumin, and sample size, subgroup analyses were conducted to detect potential sources of heterogeneity. Sensitivity analysis was conducted to examine the impact of one individual study removal on the pooled effect size. Publication bias was formally assessed using funnel plots and the Egger test if the number of included datasets was ten or higher; otherwise, only Begg's test was reported. STATA version 16.0 was used to carry out all statistical analyses (Stata Corporation, College Station, TX, US). *P* value less than 0.05 was considered significant.

## 3. Results

### 3.1. Selected Studies and Systematic Review

**Figure 1:**
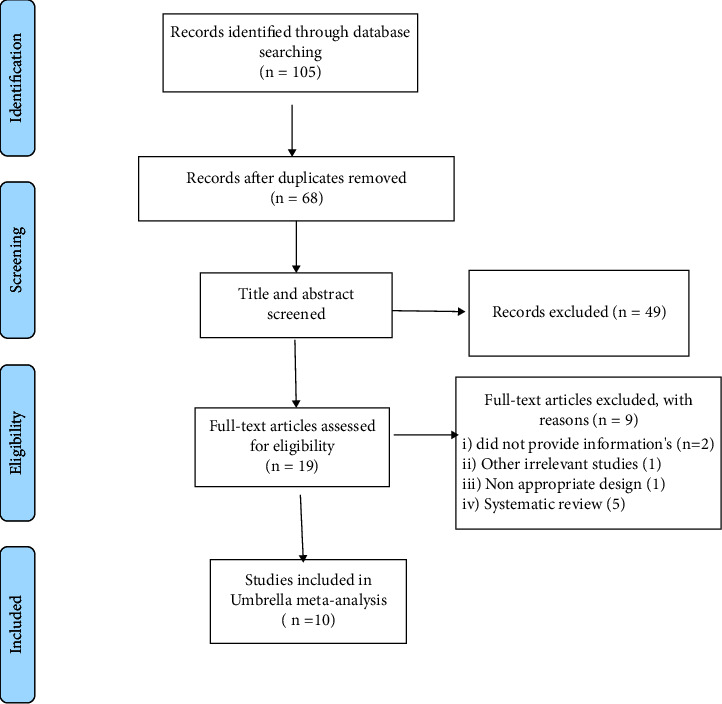
Flow diagram of study selection.

The flow diagram of the literature search process is demonstrated in Figure 1. Based on a systematic search of electronic databases, 105 articles were found. Upon removing duplicate articles and careful screening based on titles and abstracts, 19 articles were included, among which nine articles were excluded after full-text review. Overall, 10 articles met the inclusion criteria to be eligible for the umbrella meta-analysis. The characteristics of the included studies are reported in Table 1. The mean age of participants was between 29 and 52 years. The included studies were conducted from 2013 to 2021 in Iran [24–29], United States [30, 31], Australia [32], and Italy [33]. Curcumin was administered in a wide range of doses from 300 to 1900 mg, lasting from 4.5 to 10.5 weeks. Cochrane risk of bias tool and Jadad scores were used for quality assessment. The quality of the eligible RCTs in the meta-analyses is summarized in Table 1.

### 3.2. Assessment of Risk of Bias

According to AMSTAR 2, out of ten meta-analyses included in the current umbrella review, three and seven articles have high- and moderate-quality, respectively. The results of quality assessment of meta-analyses are reported in Table 2.

### 3.3. The Effects of Curcumin on CRP Levels

**Figure 2:**
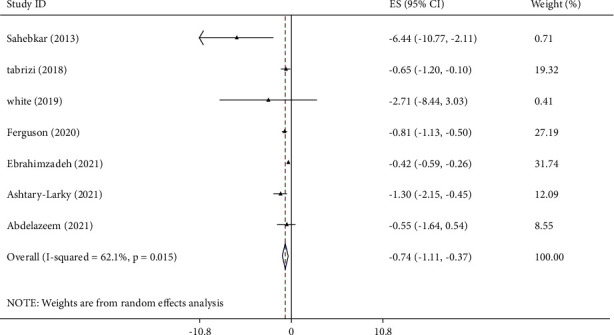
Mean difference and 95% CIs presented in forest plot of the studies on the effects of curcumin supplementation on CRP levels.

Overall, seven meta-analyses on 3,271 participants revealed a significant reduction in CRP following curcumin supplementation (Figure 2). There was a significant between-study heterogeneity (I^2^ = 62.1%, *p*=0.015). Thus, subgroup analysis was performed based on various variables. The mean age, sample size, intervention duration, and dose were potential sources of between-study heterogeneity (Table 3). Subgroup analysis showed that subjects with >45 years of age and a sample size >300 contributed to a greater decrease in the CRP level (Table 3). Sensitivity analysis revealed that the pooled results on the effects of curcumin supplementation on CRP levels did not depend on one particular study. No significant publication bias was using Begg's test (*p*=0.452). The quality of evidence for CRP was rated moderate (Table 4).

### 3.4. The Effects of Curcumin on IL-6 Levels

**Figure 3:**
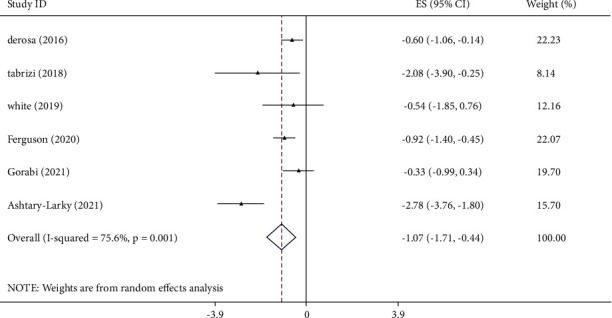
Mean difference and 95% CIs presented in forest plot of the studies on the effects of curcumin supplementation on IL-6 levels.

The effect of curcumin on IL-6 levels was reported in six studies on 2,972 individuals. The analysis showed a significant decrease in IL-6 levels by curcumin supplementation (Figure 3). The amount of heterogeneity was high (I^2^ = 75.6%, *p* < 0.001), so that mean age, duration, intervention dose, and sample size were detected as the sources of it (Table 3). Curcumin supplementation ≤700 mg/day among subjects with age 45 years or younger, intervention duration ≤7 weeks, and sample size ≤300 contributed to a greater decrease in IL-6 level (Table 3). Sensitivity analysis demonstrated no evidence of a significant effect of a single study on the overall effect size. There was no significant publication bias by Begg's test (*p*=0.452). Using the GRADE tool, the overall quality of evidence for TNF-*α* was classified as moderate (Table 4).

### 3.5. The Effects of Curcumin on TNF-*α* Levels

**Figure 4:**
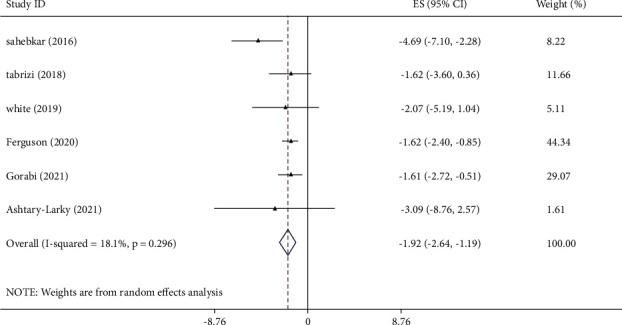
Mean difference and 95% CIs presented in forest plot of the studies on the effects of curcumin supplementation on TNF-*α* levels.

Pooled data from six studies with 3,224 participants revealed a significant lowering effect of curcumin on TNF-*α* levels (Figure 4). There was no significant heterogeneity between studies (I^2^ = 18.1%, *p*=0.296). Subgroup analysis showed a more prominent effect of curcumin supplementation on TNF-*α* levels in the sample size >300, mean age >45 years, intervention duration >7-week, and dose >700 mg/day (Table 3). Sensitivity analysis revealed that no special study affected significantly the pooled effect size. The results of Begg's test were not significant for detecting publication bias (*p*=0.060). The GRADE tool rated the overall quality of evidence for IL-6 as moderate quality (Table 4).

## 4. Discussion

### 4.1. Summary of Study Objectives and Procedures

In the present umbrella review, we consider the results of 10 systematic review meta-analyses of clinical trials that have studied the effects of curcumin supplementation on inflammatory factors including CRP, IL-6, and TNF-*α* levels. Most of the included systematic reviews 60%) have been published during three year ago (2019–2022). Most of the included studies reported the weighted mean difference (WMD) as the effect size. In the present study, the quality assessment of systematic reviews was assessed using AMSTAR 2. Most of the included studies 70%) in the final analysis had moderate quality, and 30% of the studies were scored as high quality (Table 2). Failure to comply with the PICO strategy in defining research questions, the possibility of conflicts of interest due to not reporting of financial sources, failure to consider high heterogeneity in the interpretation of results that can lead to less accurate conclusion, and failure to examine the small study effect in their analysis that can lead to less reliable results were the major flaws in some studies, resulting in their moderate quality.

### 4.2. Evaluation of Current Evidence

Overall, curcumin supplementation had a significant effect on CRP, IL-6, and TNF-*α* levels; however, there was a high level of heterogeneity in pooled CRP and IL-6 levels. Therefore, results on TNF-*α* were more reliable due to low heterogeneity. However, sources of high heterogeneity were determined using subgroup analysis, with age, study duration, sample size, and administered dose being possible sources. Therefore, the detected heterogeneity was related to clinical and methodological issues, not statistical problems resulting in high reliability of the results. This means that these subgroups had a different true effect. In terms of mean age, subgroup analysis showed that curcumin supplementation in people who are older than 45 years had a significant decreasing effect on inflammatory biomarkers in comparison with younger participants. As oxidative imbalance and inflammation increase with aging, more improving effect of curcumin on inflammation in this subgroup was not a surprising finding. According to this promising finding, curcumin can be considered as a useful agent for longevity through the reduction of oxidative stress [34]. In terms of sample size, subgroup analysis based on sample size indicated that both sample sizes ≤300 and >300 had sufficient power to detect significant results. Therefore, our results can be reliable. Subgroup analysis based on dose and duration revealed that the effect of curcumin on inflammation was not dose- or time-dependent. In fact, both high and/or low doses, and long- and/or short-term curcumin supplementation can have anti-inflammatory effects. According to the US Food and Drug Administration (FDA) report, curcumin has been considered as “Generally Recognized as Safe” (GRAS) even at doses between 4000 and 8000 mg/day [35, 36]. The bioavailability of curcumin is poor in the body, due to low absorption, rapid metabolism, limited tissue distribution, and short half-life [37]. Therefore, some studies have used some compounds such as piperine to increase the bioavailability of curcumin [38, 39]. The insignificant effect of curcumin on CRP in >700 mg/day doses and ≤7-week durations may be related to the study of Sahebkar et al. [27] who focused on the effect of curcuminoids on CRP level with characteristics of the mentioned subgroups [27]. This focus on only the type of curcuminoid supplement could potentially lead to high heterogeneity compared to other studies that include the investigations on curcumin/curcuminoids. Curcuminoids consist of three compounds including curcumin, demethoxycurcumin, and bisdemethoxycurcumin [11]. Our study population included people with NAFLD, dyslipidemia, metabolic syndrome, obesity, osteoarthritis, and migraine. The beneficial effect of curcumin on various health conditions in some studies shows that the anti-inflammatory effect of curcumin is not dependent on the disease. However, due to the diversity of diseases, subgroup analysis was not possible. As a result, no definite conclusions can be drawn. In terms of gender, participants in most of the included studies were of both genders. Therefore, it can be concluded that curcumin has anti-inflammatory effects on both genders.

Curcumin has a polyphenol nature; therefore, its anti-inflammatory mechanisms may be due to its antioxidant properties. Many properties of curcumin such as antioxidant, anti-inflammatory, antimicrobial, and antimutagenic attribute to the presence of hydroxyl and methoxy groups in the curcumin structure [40]. One of the most important factors in regulating innate immunity, cell proliferation, cell survival, and inflammation is nuclear factor-kappa-B (NF-*κ*B). This transcription factor exists in an inactive state in the cytoplasm; however, the activated form translocates from the cytoplasm to the nucleus [41]. It has been reported that curcumin can suppress NF-*κ*B activation as a proinflammatory transcription factor and prevent its translocation, which contribute to an inhibition of gene expression of several inflammatory cytokines such as IL-6, IL-2, and TNF-*α* [36, 42]. Curcumin suppresses the I*κ*B kinase (IKK) signaling complex responsible for I*κ*B phosphorylation, thereby inhibiting NF-*κ*B activation [43]. H_2_O_2_ is one of the molecules produced during oxidative stress and directly activates NF-*κ*B [44]. Curcumin can also inhibit NF-*κ*B activation by reducing oxidative stress and free radicals due to its antioxidant characteristics [45]. Also, it has been reported that dietary polyphenols have a beneficial effect on nuclear factor erythroid 2-related factor 2 (Nrf2) and internal antioxidant status, through which they play the important roles in balancing oxidative stress. Nrf-2 in an inactive form binds to the Kelch-like ECH-associated protein 1 (Keap1) and is present in the cytoplasm [46]. Dietary polyphenols interact with the amino acid cysteine and cause the dissociation of Keap1 from Nrf2, thereby translocating Nrf2 to the nucleus. Subsequently, Nrf-2 binds to antioxidant response elements (AREs) that are responsible for stimulating the expression of genes involved in the antioxidant system such as superoxide dismutase (SOD), catalase (CAT), and glutathione peroxidase (GPx) [47, 48]. Curcumin may also act as a TNF-*α* blocker by connecting directly to TNF-*α* [49]. Furthermore, curcumin is able to inhibit the expression and release of proinflammatory factors through interacting with receptors and signaling pathways [50]. In addition, curcumin can inhibit cyclooxygenase-2 gene expression, which is one of the main enzymes involved in inflammatory pathways [51]. In addition to the anti-inflammatory effects of curcumin mentioned above, which can affect CRP levels as a general indicator of inflammation, curcumin also interacts with signaling pathways leading to CRP production. Zhang et al. reported that curcumin reduces CRP production in vascular smooth muscle cells by inhibiting the reactive oxygen species-ERK1/2 pathway [52]. ERK1/2 (extracellular signal-regulated protein kinase) has a pivotal role in delivering extracellular signals to the nucleus [53]. Based on in silico approaches, it was found that curcumin also has a direct interaction with CRP through binding to GLN 150 and ASP 140 sites [54].

To our knowledge, the present study is the first umbrella review of meta-analysis studies on clinical trials investigating the effects of curcumin supplementation on inflammation. All included studies had a moderate to high quality score based on the AMSTAR 2 tool. Furthermore, subgroup analysis was conducted to adjust for heterogeneity and evaluate the effect of curcumin in different conditions.

This study has some limitations that must be mentioned. The first is the repetition of some studies in different meta-analyses, which can affect the final result. However, further assessments showed that the repeated studies did not have much weight on the final result. Second, the interpretation of our results must be with precaution due to high between-study heterogeneity. However, possible sources of heterogeneity were studied by subgroup analysis. Third, due to the wide range of diseases that systematic reviews incorporated in their studies, subgroup analysis based on the study population was not possible. Fourth, only published systematic review and meta-analysis studies were considered for the final analysis in the present umbrella review. This approach might lead to the missing of a number of published clinical trials. The current umbrella systematic review and meta-analysis had several strengths. First, a comprehensive subgroup analysis was conducted to find possible sources of heterogeneity and bias. Second, the data quality of the current study was thoroughly evaluated to present reliable results. Third, the GRADE tool was employed to assess the certainty of the evidence.

## 5. Conclusion

The present study shows that curcumin has reducing effects on IL-6, CRP, and TNF-*α* levels. Older people benefited more from curcumin supplementation. Therefore, curcumin can be considered as a useful agent for longevity through decreasing oxidative stress. The anti-inflammatory effect of curcumin was not dose- and time-dependent.

## Figures and Tables

**Table 1:** Study characteristics of included studies.

Citation (first author et al., year)	No. of studies in meta-analysis	Country	No. of participants in meta-analysis	Mean age (years)	Duration of intervention	Dose (mg/daily)	Quality assessment scale and outcome
Ebrahimzadeh et al. 2021 [24]	6	Iran	167	609	10.5 weeks	583	Yes (Cochrane) 3/6 high
Ashtary-Larky et al. 2021 [25]	5	Iran	298	51	10 weeks	100	Yes (Cochrane) 4/5 high
Gorabi et al. 2021 [26]	17	Iran	1033	49	8 weeks	900	Yes (Jadad score) NR
Sahebkar et al. 2014 [27]	6	Iran	342	48	4.5 weeks	1900	Yes (Cochrane) 4/6 high
Sahebkar et al. 2016 [28]	8	Iran	449	52	7 weeks	1014	Yes (Cochrane) 2/8 high
Tabrizi et al. 2018 [29]	11	Iran	797	NR	7 weeks	950	Yes (Cochrane) 10/11 high
Abdelazeem et al. 2021[30]	2	USA	81	29	7 weeks	300	Yes (Cochrane) 1/2 high
White et al. 2019 [31]	8	USA	654	46	8 weeks	686	Yes (Cochrane) 2/8 high
Ferguson et al. 2020 [32]	19	Australia	1440	49	8 weeks	512	Yes (Cochrane) 7/19 high
Derosa et al. 2016 [33]	9	Italy	609	51.5	9 weeks	1422	Yes (Cochrane) 3/9 high

NR, not reported.

**Table 2:** Results of assessing the methodological quality of meta-analysis.

Citation (first author et al., year)	Q1	Q2	Q3	Q4	Q5	Q6	Q7	Q8	Q9	Q10	Q11	Q12	Q13	Q14	Q15	Q16	Quality assessment
Ebrahimzadeh et al. 2021[24]	No	Yes	Yes	Partial yes	Yes	Yes	Yes	Yes	Yes	No	Yes	Yes	Yes	Yes	No	Yes	Moderate
Ashtary-Larky et al. 2021[25]	No	Yes	Yes	Yes	Yes	Yes	Yes	Yes	Yes	No	Yes	No	Yes	Yes	Yes	Yes	High
Gorabi et al. 2021[26]	No	Yes	Yes	Partial yes	No	Yes	Yes	Partial yes	Yes	No	Yes	No	Yes	Yes	Yes	Yes	Moderate
Sahebkar et al. 2014 [27]	No	Yes	Yes	Partial yes	Yes	Yes	Partial yes	Yes	No	Yes	Yes	Yes	Yes	Yes	No	Yes	Moderate
Sahebkar et al. 2016 [28]	No	Partial yes	Yes	Partial yes	Yes	Yes	Yes	Yes	Yes	No	Yes	Yes	Yes	Yes	Yes	No	Moderate
Tabrizi et al. 2018 [29]	No	Yes	Yes	Partial yes	Yes	Yes	Yes	Yes	Yes	Yes	Yes	Yes	Yes	Yes	No	Yes	High
Abdelazeem et al. 2021[30]	No	Yes	Yes	Partial yes	Yes	Yes	Partial yes	Yes	Yes	No	Yes	Yes	Yes	No	Yes	Yes	Moderate
White et al. 2019 [31]	No	Yes	Yes	Partial yes	Yes	Yes	Yes	No	Yes	No	Yes	Yes	Yes	Yes	No	Yes	Moderate
Ferguson et al. 2020 [32]	Yes	Partial yes	Yes	Partial yes	Yes	Yes	Yes	Yes	Yes	Yes	Yes	Yes	Yes	Yes	Yes	Yes	High
Derosa et al. 2016 [33]	No	Partial yes	Yes	Partial yes	Yes	Yes	Yes	Yes	Yes	No	Yes	Yes	Yes	Yes	Yes	No	Moderate

^*∗*^(1) Did the research questions and inclusion criteria for the review include the components of PICO? (2) Did the report of the review contain an explicit statement that the review methods were established prior to the conduct of the review and did the report justify any significant deviations from the protocol? (3) Did the review authors explain their selection of the study designs for inclusion in the review? (4) Did the review authors use a comprehensive literature search strategy? (5) Did the review authors perform study selection in duplicate? (6) Did the review authors perform data extraction in duplicate? (7) Did the review authors provide a list of excluded studies and justify the exclusions? (8) Did the review authors describe the included studies in adequate detail? (9) Did the review authors use a satisfactory technique for assessing the risk of bias (RoB) in individual studies that were included in the review? (10) Did the review authors report on the sources of funding for the studies included in the review? (11) If meta-analysis was performed, did the review authors use appropriate methods for statistical combination of results? (12) If meta-analysis was performed, did the review authors assess the potential impact of RoB in individual studies on the results of the meta-analysis or other evidence synthesis? (13) Did the review authors account for RoB in individual studies when interpreting/discussing the results of the review? (14) Did the review authors provide a satisfactory explanation for, and discussion of, any heterogeneity observed in the results of the review? (15) If they performed quantitative synthesis, did the review authors carry out an adequate investigation of publication bias (small study bias) and discuss its likely impact on the results of the review? (16) Did the review authors report any potential sources of conflicts of interest, including any funding they received for conducting the review? Each question was answered with “yes,” “partial yes” or “no.” When no meta-analysis was done, question 11, 12, and 15 were answered with “no meta-analysis conducted.”

**Table 3:** Subgroup analyses for the effects of curcumin supplementation on inflammatory biomarkers.

	Effect size (*n*)	ES (95% CI)^1^	*P*-within^2^	*I* ^2^ (%)^3^	*P*-heterogeneity^4^
*Curcumin supplementation on CRP levels*					
*Age (years)*					
≤45	2	−0.42 (−0.59, −0.26)	<0.001	0	0.817
>45	4	−1.34 (−2.36, −0.33)	0.009	61.6	0.050
NR	1	−0.65 (−1.20, −0.10)	0.021	—	—

*Intervention duration (week)*					
≤7	2	−3.11 (−8.83, 2.61)	0.287	85	0.010
>7	5	−0.68 (−0.98, −0.37)	<0.001	54	0.069

*Sample size*					
≤300	4	−0.65 (−1.15, −0.16)	0.009	34.7	0.204
>300	3	−0.90 (−1.64, −0.15)	0.018	70.6	0.033
*Dose (mg/day)*					
≤700	5	−0.68 (−1.02, −0.34)	<0.001	53.1	0.074
>700	2	−3.13 (−8.75, 2.49)	0.275	85.2	0.009

*Curcumin supplementation on serum IL-6 levels Age (years)*					
≤45	2	−0.37 (−0.97, 0.22)	0.217	0	0.779
>45	3	**−1.32 (−2.27, −0.37)**	0.006	87.2	<0.001
NR	1	−2.08 (−3.90, −0.25)	0.025	—	—

*Intervention duration (weeks)*					
≤7	2	−1.15 (−2.06, −0.25)	0.012	30.9	0.229
>7	4	−1.03 (−1.99, −0.07)	0.036	83.8	<0.001

*Sample size*					
≤300	2	−2.62 (−3.49, −1.76)	<0.001	0	0.508
>300	4	−0.66 (−0.95, −0.38)	<0.001	0	0.526

*Dose (mg/day)*					
≤700	4	−1.54 (−2.61, −0.46)	0.005	77.1	0.004
>700	2	−0.51 (−0.89, −0.13)	0.008	0	0.517

*Curcumin supplementation on serum TNF-α levels Age (years)*					
≤45	3	−1.64 (−2.27, −1.01)	<0.001	0	0.880
>45	2	−3.57 (−6.12, −1.03)	0.006	41.3	0.192
NR	1	−1.62 (−3.60, 0.36)	0.109	—	—

*Intervention duration (weeks)*					
≤7	3	−1.65 (−2.35, −0.94)	<0.001	0	0.963
>7	3	−2.91 (−5.28, −0.55)	0.016	62.3	0.071

*Sample size*					
≤300	2	−1.78 (−3.65, 0.09)	0.062	0	0.631
>300	4	−2.09 (−3.12, −1.05)	<0.001	48.9	0.118

*Dose (mg/day)*					
≤700	3	−1.67 (−2.42, −0.93)	<0.001	0	0.853
>700	3	−2.40 (−4.10, −0.71)	0.005	63	0.067

^1^Obtained from the random-effects model, ^2^refers to the mean (95% CI), ^3^inconsistency, percentage of variation across studies due to heterogeneity, ^4^obtained from the *Q*-test. Abbreviation: ES: effect size; CI: confidence interval; NR: not reported.

**Table 4:** Assessing the quality of evidence using the GRADE approach.

Outcome measures	Summary of findings		Quality of evidence assessment (GRADE)					
	No. of patients (meta-analysis)	Effect size^*∗*^ (95% CI)	Risk of bias^a^	Inconsistency^b^	Indirectness^c^	Imprecision^d^	Publication bias^e^	Quality of evidence^f^
Inflammatory biomarkers								
CRP	3,271 (7)	−0.74 (−1.11, −0.37)	Not serious	Not serious	Serious	Not serious	Not serious	Moderate
TNF-*α*	3,224 (6)	−1.92 (−2.64, −1.19)	Not serious	Not serious	Serious	Not serious	Not serious	Moderate
IL-6	2,972 (6)	−1.07 (−1.71, −0.44)	Not serious	Not serious	Serious	Not serious	Not serious	Moderate

CRP: C-reactive protein; TNF: tumor necrosis factor; IL-6: interleukin-6. (a) Risk of bias based on the according to AMSTAR results. (b) Downgraded if there was a substantial unexplained heterogeneity (I^2^ > 50%, *P* < 0.10) that was unexplained by meta-regression or subgroup analyses. (c) Downgraded if there were factors present relating to the participants, interventions, or outcomes that limited the generalizability of the results. Participants of the included studies were from different health conditions. (d) Downgraded if the 95% confidence interval (95% CI) crossed the minimally important difference (MID) for benefit or harm. MIDs used for each outcome were 3.16 mg/l for CRP, 7.9 pg/ml for TNF-*α*, and 2 pg/ml for IL-6. (e) Downgraded if there was evidence of publication bias using funnel plot. (f) Since all included studies were meta-analysis of randomized controlled trials, the certainty of the evidence was graded as high for all outcomes by default, and then downgraded based on prespecified criteria. Quality was graded as high, moderate, low, or very low.

## Data Availability

No data were used to support this study.

## Supplementary Materials

Supplementary Materials Table 1: electronic search strategy.
