# Supplementary material for: Profiling Inflammatory Biomarkers following Curcumin Supplementation: An Umbrella Meta-Analysis of Randomized Clinical Trials
Source: Evid Based Complement Alternat Med. 2023 Jan 16;2023:4875636. doi: 10.1155/2023/4875636 (PMC9870680; doi:10.1155/2023/4875636)
Supplement: Supplementary Materials — Table 1: electronic search strategy. [file 4875636.f1.doc]

**Profiling Inflammatory Biomarkers Following Curcumin Supplementation: An Umbrella of Meta-analysis of Randomized Clinical Trials**

**Table S1** Electronic search strategy

| **PUBMED**  **Descriptors** |
| --- |
| ("curcumin"[MeSH Terms] OR ("curcumin"[Title/Abstract] OR "curcuminoid"[Title/Abstract] OR "turmeric"[Title/Abstract]) **AND** ("Inflammation"[MeSH Terms] OR "Inflammation"[Title/Abstract] OR "c reactive protein"[MeSH Terms] OR "c reactive protein"[Title/Abstract] OR "crp"[Title/Abstract] OR "hs-crp"[Title/Abstract] OR "high sensitivity crp"[Title/Abstract] OR "high sensitivity c reactive protein"[Title/Abstract] OR "tumor necrosis factor alpha"[MeSH Terms] OR "tumor necrosis factor alpha"[Title/Abstract] OR "tnf-alpha"[Title/Abstract] OR "tnf-alpha"[Title/Abstract] OR "Interleukin-6"[MeSH Terms] OR "Interleukin-6"[Title/Abstract] OR "IL-6"[Title/Abstract] OR "Interleukin-6"[Title/Abstract]) **AND** ("systematic review"[Publication Type] OR "meta-analysis"[Title/Abstract]) |
